# Impact of preferred surgical modality on surgeon wellness: a survey of workload, physical pain/discomfort, and neuromusculoskeletal disorders

**DOI:** 10.1007/s00464-023-10485-0

**Published:** 2023-10-23

**Authors:** Hamid Norasi, M. Susan Hallbeck, Enrique F. Elli, Matthew K. Tollefson, Kristi L. Harold, Raymond Pak

**Affiliations:** 1 Robert D. and Patricia E. Kern Center for the Science of Healthcare Delivery, Mayo Clinic, Rochester, MN USA; 2 Division of Health Care Delivery Research, Mayo Clinic, Rochester, MN USA; 3 Department of Surgery, Mayo Clinic, Rochester, MN USA; 4 Department of Surgery, Mayo Clinic, Jacksonville, FL USA; 5 Department of Urology, Mayo Clinic, Rochester, MN USA; 6 Department of Surgery, Mayo Clinic, Phoenix, AZ USA; 7 Department of Urology, Mayo Clinic, Jacksonville, FL USA

**Keywords:** Robotic surgery, Endoscopic surgery, Laparoscopic surgery, Open surgery, Ergonomics, Da Vinci surgical system

## Abstract

**Background:**

We compared surgeons’ workload, physical discomfort, and neuromusculoskeletal disorders (NMSDs) across four surgical modalities: endoscopic, laparoscopic, open, and robot-assisted (da Vinci Surgical Systems).

**Methods:**

An electronic survey was sent to the surgeons across an academic hospital system. The survey consisted of 47 questions including: (I) Demographics and anthropometrics; (II) The percentage of the procedural time that the surgeon spent on performing each surgical modality; (III) Physical and mental demand and physical discomfort; (IV) Neuromusculoskeletal symptoms including body part pain and NMSDs.

**Results:**

Seventy-nine out of 245 surgeons completed the survey (32.2%) and 65 surgeons (82.2%) had a dominant surgical modality: 10 endoscopic, 15 laparoscopic, 26 open, and 14 robotic surgeons. Physical demand was the highest for open surgery and the lowest for endoscopic and robotic surgeries, (all *p* < 0.05). Open and robotic surgeries required the highest levels of mental workload followed by laparoscopic and endoscopic surgeries, respectively (all *p* < 0.05 except for the difference between robotic and laparoscopic that was not significant). Body part discomfort or pain (immediately after surgery) were lower in the shoulder for robotic surgeons compared to laparoscopic and open surgeons and in left fingers for robotic surgeons compared to endoscopic surgeons (all *p* < 0.05). The prevalence of NMSD was significantly lower in robotic surgeons (7%) compared to the other surgical modalities (between 60 and 67%) (all *p* < 0.05).

**Conclusions:**

The distribution of NMSDs, workload, and physical discomfort varied significantly based on preferred surgical approach. Although robotic surgeons had fewer overall complaints, improvement in ergonomics of surgery are still warranted.

**Graphical abstract:**

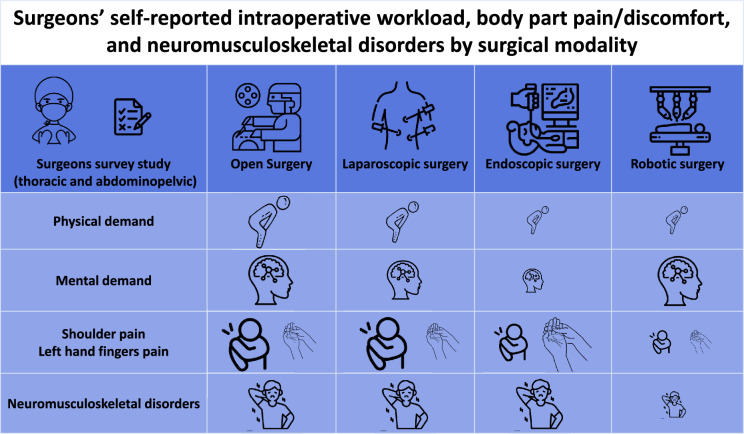

Advances in surgical technology and technique have improved patient outcomes and allowed for more complex minimally invasive procedures. Despite these innovations, the prevalence of neuromusculoskeletal disorders (NMSDs), pain, and physical discomfort reported by surgeons have increased (e.g., degenerative spine diseases) in the past decade [1, 2]. This “impending epidemic” [3] negatively affects surgeon well-being, daily life (e.g., sleep) [4] and career longevity and productivity. Previous studies have demonstrated almost 50% of surveyed surgeons feel that physical discomfort could negatively impact surgery performance and restrict career longevity [4, 5]. In response to NMSDs, pain and physical discomfort, surgeons have reported early retirement, burnout, leaving surgical careers, restricted practices, and going on short- or long-term disability [1, 4–7]. These outcomes may further confound the widening gap of surgeon supply and patient demand [8, 9].

Due to the increasing prevalence of work-related injuries reported by surgeons, there is a critical need for better ergonomic understanding and interventions in the operating room (OR). While previous studies have explored potential ergonomic interventions such as physical exercise outside the OR [10], intraoperative stretching microbreaks [11–14] and more recently passive exoskeletons [15–17], there still remain many factors contributing to this problem. One unanswered question is: does surgeon preference for a surgical modality (e.g., open, laparoscopic, endoscopic, robot-assisted) affect the incidence of NMSDs, pain, and physical discomfort? As each modality is associated with a different operative posture and orchestration of instrumentation, it warrants a further understanding to determine if any of these surgical modalities are ergonomically protective or harmful for surgeons. Additionally, it will help refine potential ergonomic interventions within the preferred surgical modality to enhance feasibility, applicability, and effectiveness.

Although previous studies have investigated the ergonomics and NMSDs over different surgical modalities, a comprehensive study that compares different surgical modalities across several surgical specialties is lacking. A systematic review and meta-analysis of surgical ergonomics reported minimally invasive surgeries (MIS) to be associated with higher odds of body part pain (e.g., neck, hands), fatigue and numbness [2]; however, there are other studies that question these findings [18–21]. For example, an objective evaluation of surgeons’ postural exposure found open surgeries to be more stressful than laparoscopic surgeries [19]. Most of these studies are performed by various surgical societies or over a limited speciality group, which highlights the need for a more comprehensive study with more granular investigations by dividing MIS to laparoscopic, endoscopic, and robot-assisted modalities.

This is of particular importance since laparoscopic surgery is typically conducted from a standing position, while robotic and endoscopic surgery is typically conducted from a seated position. In recent studies, robot-assisted surgery has been found to be more physically beneficial to surgeons when compared to open [21–23] and laparoscopic [21–24] approaches; however, the results are not always affirmative [25, 26]. Neuromusculoskeletal problems and physical strain associated with robot-assisted approaches require more investigation of ergonomics for this surgical modality as well [21–23, 27].

The main goal of this study was to evaluate and compare surgeons’ workload, physical discomfort, and NMSDs among four surgical modalities (endoscopic, laparoscopic, open, and robot-assisted). Our secondary goal was to explore if surgeons primarily performing robot-assisted surgery (specifically the da Vinci Xi or SP Surgical Systems) had a different NMSD profile when compared to other surgical modalities. The study focused on thoracic and abdominopelvic surgeons because they typically employ several surgical modalities in their surgical practice. In favor of simplicity, “robotic surgery” has been used instead of robot-assisted surgery in the following sections.

## Methods

### Participants and experimental procedure

This study was approved by the Institutional Review Board (IRB) of Mayo Clinic, Rochester, Minnesota. The inclusion criteria covered all urologic, gynecologic, thoracic, and general (including breast, colorectal, hepato-pancreato-biliary, and bariatric) surgeons across all geographically distinct parts of this academic hospital system (midwest hospital, southeast hospital, southwest hospital, and a number of health system hospitals) in the United States. An electronic survey using Qualtrics (Qualtrics, Provo, UT) was sent to the surgeons through their institutional email address with three reminders. The survey consisted of 47 questions and included: (I) Demographics and anthropometrics (e.g., age, gender, stature, weight, and hand dominance); (II) The percentage of the procedural time that the surgeon spent on performing each of the surgical modalities (endoscopic, laparoscopic, open, and robotic (specifically the da Vinci Xi or SP Surgical Systems) surgeries) on a typical surgical day; (III) Questions that asked about physical and mental demand (modified from Surgery Task Load Index (SURG-TLX) [28]) and physical discomfort by surgical modality. If the surgeon spent 0% of their surgical time utilizing a specific surgical modality, they were not asked these questions for that surgical modality; (IV) Questions on neuromusculoskeletal symptoms including physical discomfort, body part pain (modified from standardized Nordic musculoskeletal questionnaires NMSQ [29]), and NMSDs. These questions were asked generally, without specifying a surgical modality; (V) Questions about well-being, job satisfaction, burnout, and treatments/interventions to address neuromusculoskeletal pain and discomfort, (VI) Questions about surgical modality selection and the order of surgical cases.

### Experimental design

#### Independent variables

The independent variable in this study was the surgical modality with four disciplines of (1) endoscopic (e.g., thoracoscopy, bronchoscopy; note that vaginal procedures were not considered as endoscopic), (2) laparoscopic (e.g., laparoscopic subtotal colectomy with anastomosis), (3) open (e.g., bilateral skin sparing mastectomy), and (4) robotic (e.g., robot-assisted low anterior resection with anastomosis). The survey did not limit the definition of surgical modalities and relied on surgeons’ definition of the surgical modalities. For example, endoscopic surgeries covered a wide range of procedures from simple diagnostic endoscopies to advanced therapeutic endoscopy.

#### Dependent variables

The dependent variables in this study were the participants’ responses to the questions related to their workload, physical discomfort, and NMSDs (question groups III and IV). The data from question groups V, and VI are intended to be discussed in another paper by the same team of authors.

### Statistical analysis

No randomization strategy was performed as all potential participants were sent the electronic survey. Associations between the surgical modality and dependent variables were evaluated using two approaches.

#### Questions asked per surgical modality

The effects of modality on dependent variables that were recorded per surgical modality were evaluated using non-parametric Kruskal–Wallis test and post-hoc non-parametric Wilcoxon Signed-Rank test (pairwise comparisons).

#### Questions not based on a specific surgical modality (e.g., NMSDs)

The effects of surgical modality on dependent variables that were not recorded per surgical modality were evaluated after each surgeon was allocated to a dominant surgical modality. The threshold for defining a dominant modality was the difference in the percentage of the procedural time that a surgeon spent on performing a surgical modality. If that was “at least 10% higher” than the other three modalities, the surgeon was allocated to that surgical modality as their dominant. Then, either non-parametric Kruskal–Wallis test and post-hoc non-parametric Wilcoxon Signed-Rank test (pairwise comparisons) or logistic regression and Wald based pairwise comparisons were performed (dependent on the type of the data). For all the statistical analyses in this study the significance level of 0.05 was considered. Pairwise comparisons were performed only if the main model (including the four surgical modalities) showed significant effects of the surgical modality on the studied variable (*p* < 0.05).

## Results

The electronic survey was sent to 245 thoracic and abdominopelvic surgeons across an academic hospital system. Seventy-nine surgeons completed the survey (response rate 32.2%), including 19 urologic, 22 gynecologic, 3 thoracic, and 35 general (including breast, colorectal, hepato-pancreato-biliary, and bariatric) surgeons.

The average (standard deviation (SD)) of the 79 respondent surgeons’ demographics and anthropometrics were as follows: age 46.6 (9.3) years, weight 77.7 (14.6) kg, height 174.1 (8.9) cm. Thirty-one participants (39%) were female surgeons, 72 participants (92%) were right-handed, four surgeons (5%) were left-handed, two surgeons (3%) were completely ambidextrous, and one surgeon did not respond to the hand dominance question.

**Fig. 1:**
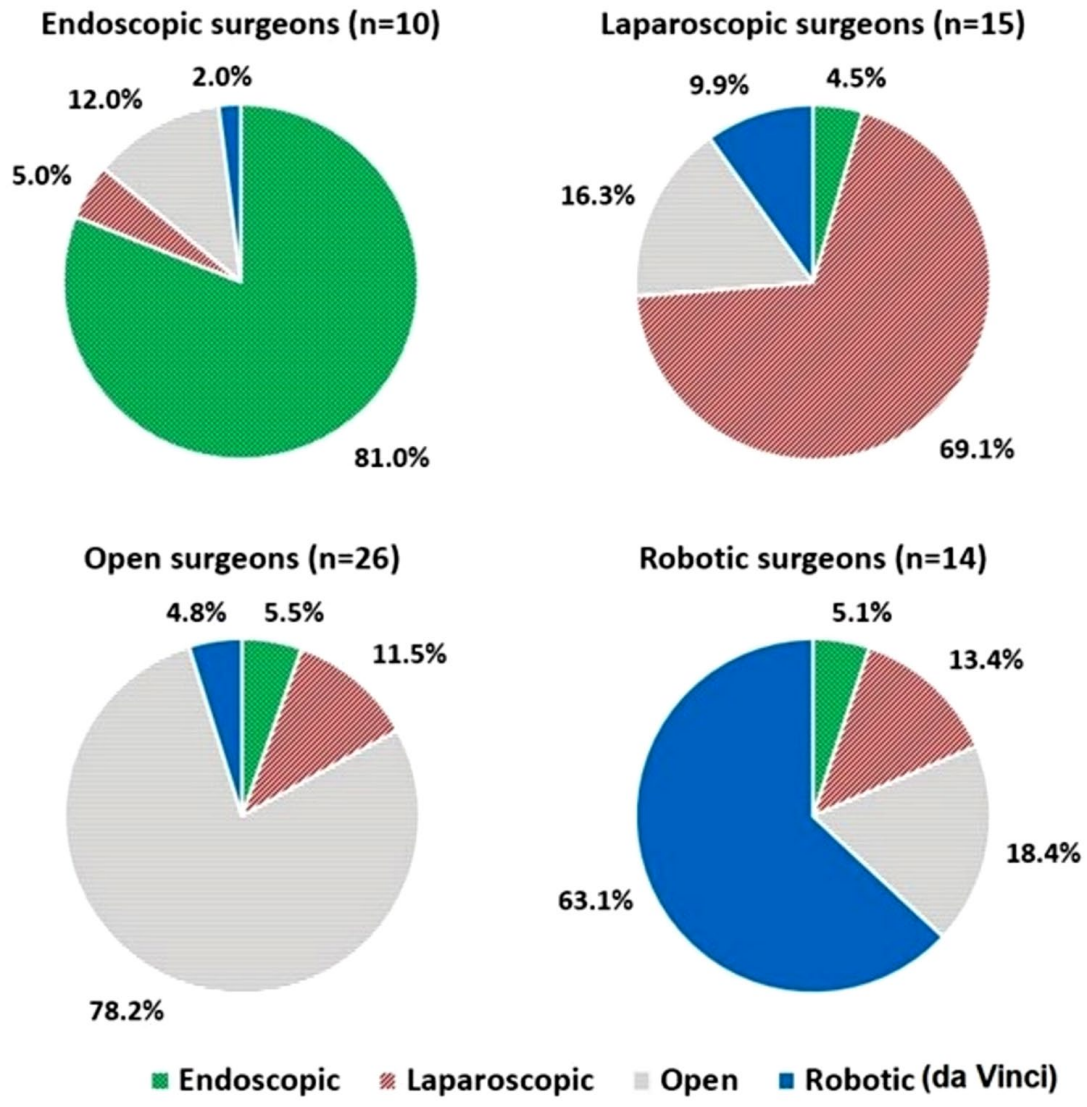
Surgeons’ dominant modality and the average percentage of time spent on the four surgical modalities. *n* number of participants

Out of the 79 respondents, 65 surgeons (82.2%) had a dominant surgical modality based on our defined threshold, while 14 surgeons did not belong to any specific surgical modality. The average (SD) of the percentage of the procedural time that a surgeon spent on their dominant surgical modality were 81.0% (21.4%) for endoscopic, 69.1% (19.8%) for laparoscopic, 78.2% (19.4%) for open, and 63.1% (12.5%) for robotic surgeons (Fig. 1).

### Questions asked by surgical modality

**Fig. 2:**
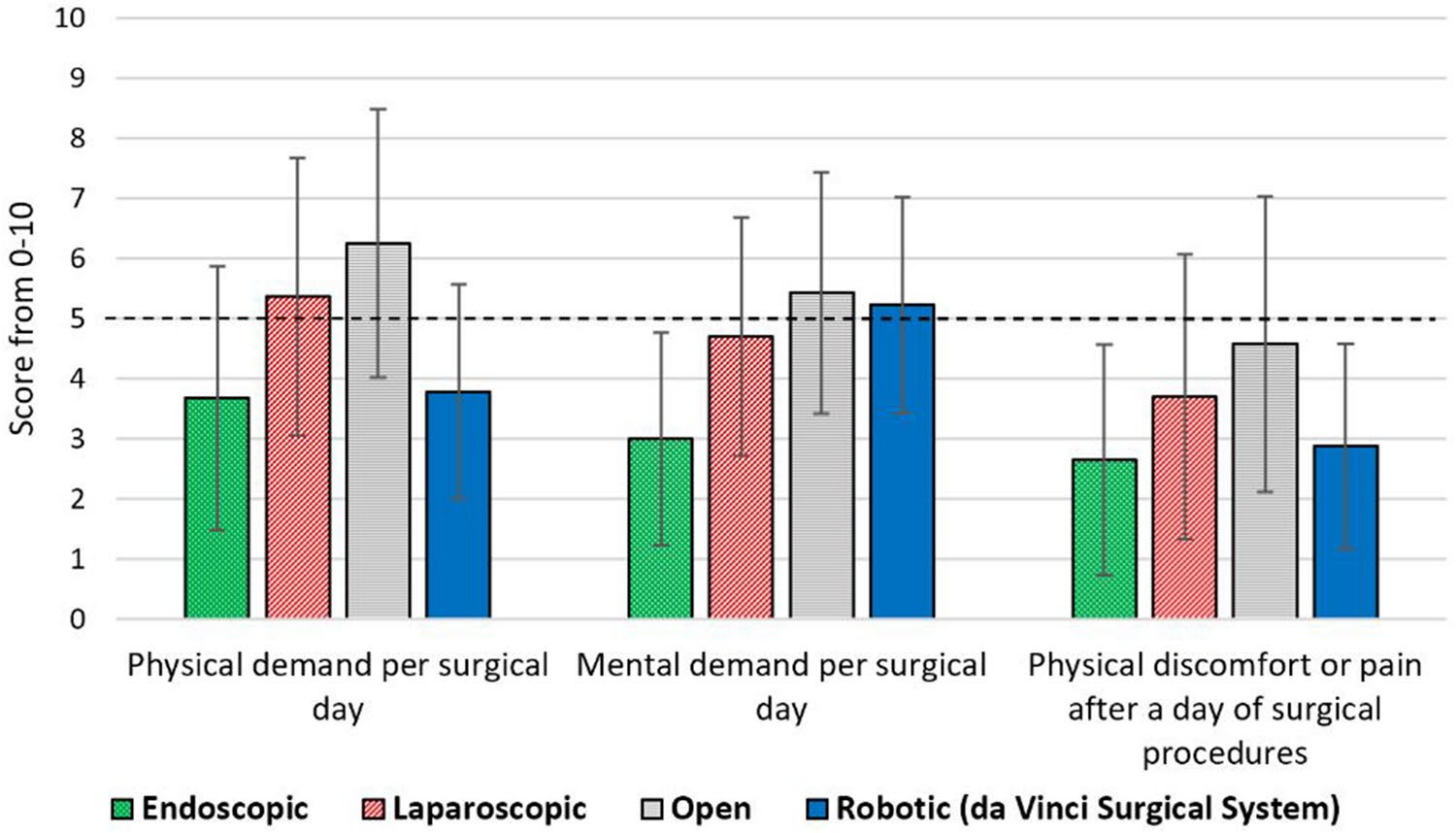
Mean and standard deviation of workload and physical pain and discomfort per surgical modalities (endoscopic, laparoscopic, open, and robotic surgeries)

These questions specifically asked about intraoperative physical demand per typical surgical day, intraoperative mental demand per typical surgical day, and physical discomfort or pain (over the last 12 months) after a full day of procedures (all rated from 0 = Not applicable to 10 = Worst imaginable). The number of the responses to these three questions varied for surgical modalities (38 for endoscopic, 53 for laparoscopic, 72 for open, and 40 for robotic questions). The results of the analysis are presented in Fig. 2 and Table 1.

**Table 1:** Associations between surgical modalities (endoscopic, laparoscopic, open, and robotic) and workload and physical discomfort

	Schematic pairwise comparisons (median)				Pairwise comparisons	*p*-value
Physical demand per surgical day	Open (7)	** A**			Open > Endoscopic	< 0.0001
	Laparoscopic (5)		**B**		Open > Laparoscopic	0.0163
	Endoscopic (4)			**C**	Open > Robotic	< 0.0001
	Robotic (3)			**C**	Laparoscopic > Endoscopic	0.0016
					Laparoscopic > Robotic	0.0008
Mental demand per surgical day	Open (6)	** A**			Open > Endoscopic	< 0.0001
	Laparoscopic (4.5)		**B**		Open > Laparoscopic	0.0336
	Endoscopic (3)			**C**	Robotic > Endoscopic	< 0.0001
	Robotic (5)	** A**	**B**		Laparoscopic > Endoscopic	< 0.0001
Physical discomfort/pain after a day of surgical procedures	Open (5)	** A**			Open > Endoscopic	0.0002
	Laparoscopic (3)		**B**		Open > Laparoscopic	0.0374
	Endoscopic (3)		**B**		Open > Robotic	0.0002
	Robotic (3)		**B**			

Additionally, there was a question about the time after the start of the operative day that the surgeon experienced discomfort or pain (attributable to the surgeries) noticed with seven levels of 1 (< 30 min) to 6 (> 6 h) while level 7 meant “no discomfort/pain”. The responses revealed no significant difference over the four surgical modalities. The median value of the chosen levels was five (4–6 h) for endoscopic and robotic surgeries and four (2–4 h) for laparoscopic and robotic surgeries.

### Questions asked generally (not asked by specified surgical modality)

**Fig. 3:**
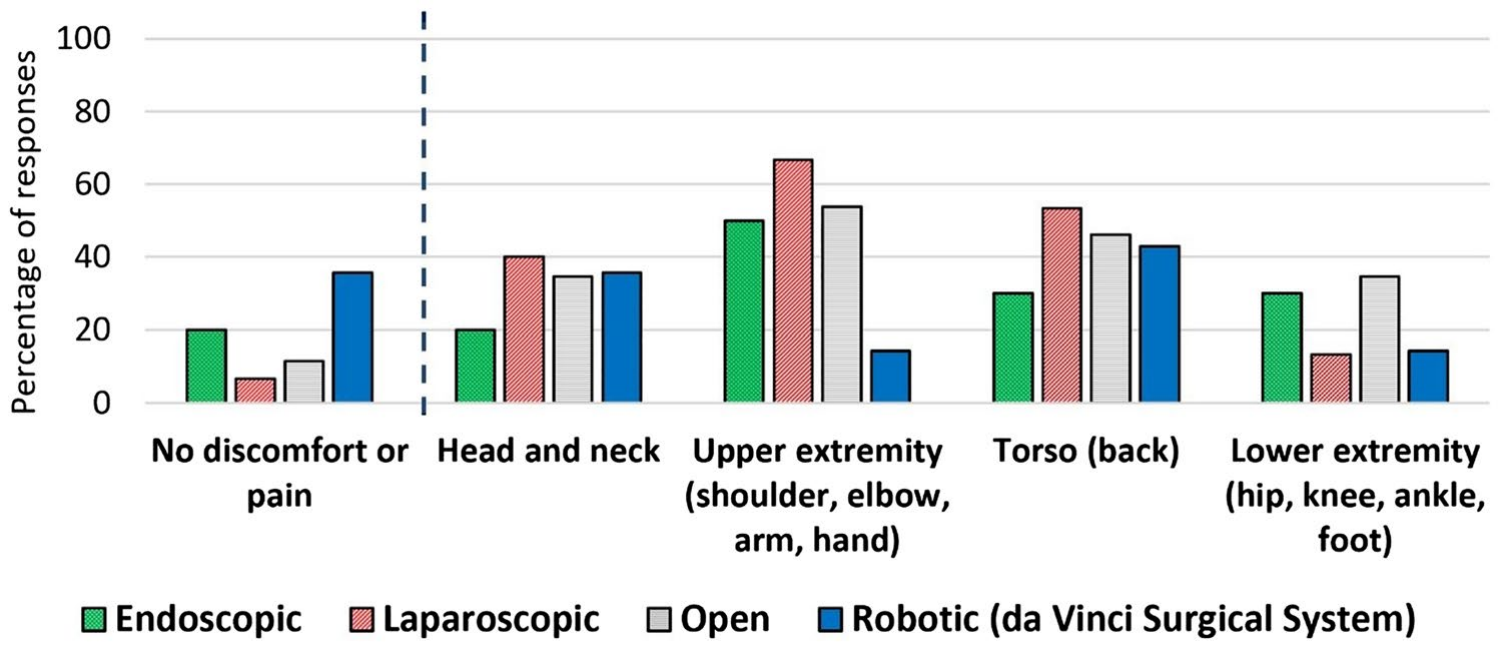
Responses to a binary question asking if the surgeon had any physical discomfort or pain in specific body regions

The following surgeon responses were not asked by surgical modality. However, to analyze, each surgeon was allocated to a dominant surgical modality (if the percentage of the procedural time they spent on performing a surgical modality was “at least 10% higher” than the other three modalities). This led to defining 65 surgeons as: 10 endoscopic, 15 laparoscopic, 26 open, and 14 robotic surgeons (Fig. 1). The surgeons were asked if they had baseline pain prior to their surgical day. There was no significant difference across the four modalities, one endoscopic (10%), five laparoscopic (33%), eleven open (42%) and two robotic (14%) surgeons responded “yes” (29% “yes” for the 65 surgeons with a dominant modality as well as for the total 79 surgeons) and they all indicated in the next question that their baseline pain was exacerbated by surgery. All surgeons were asked if they ever had or currently have neuromusculoskeletal pain. Fifty-nine percent of the 79 surgeons (regardless of modality) and 62% of the 65 surgeons with a dominant modality answered “yes” to this question. The results revealed that this problem is less prevalent among robotic surgeons compared to surgeons allocated to other dominant surgical modalities (all *p* < 0.01) (Table 2). Figure 3 presents the result of a binary question asking if the surgeon had any physical discomfort or pain in specific body regions. As presented in Table 2, the only statistically significant difference was less discomfort or pain in upper extremity in robotic surgeons compared to laparoscopic (*p* = 0.0082) and open (*p* = 0.0235) surgeons.

**Table 2:** Difference in neuromusculoskeletal symptoms by surgical modalities (endoscopic, laparoscopic, open, and robotic)

	Model *p*-value^*^	Pairwise comparisons		
		Comparison^a^ % reporting YES	*p*-value	Odds ratio^b^ CI (L–U 95%)^c^
Ever had or currently have neuromusculoskeletal pain	0.0057	Robotic (21%)/Endoscopic (80%)	0.0087	0.068 (0.009–0.508)
		Robotic (21%)/Laparoscopic (73%)	0.0082	0.099 (0.018–0.551)
		Robotic (21%)/Open (69%)	0.0067	0.121 (0.026–0.557)
Any physical discomfort or pain in upper extremity	0.0219	Robotic (14%)/Laparoscopic (67%)	0.0082	0.083 (0.013–0.526)
		Robotic (14%)/Open (54%)	0.0235	0.143 (0.027–0.770)
Any neuromusculoskeletal disorders^d^	0.0013	Robotic (7%)/Endoscopic (60%)	0.0151	0.051 (0.005–0.563)
		Robotic (7%)/Laparoscopic (67%)	0.0055	0.038 (0.004–0.384)
		Robotic (7%)/Open (62%)	0.0064	0.048 (0.005–0.426)

^a^Based on Wald test

^b^Robotic is the exposed group and the other modality is the unexposed group. “Yes” is the case and “No” is the control

^c^CI (L–U 95%) = confidence interval (lower–upper 95%)

^d^This includes at least one of the following symptoms: cervical disc issues, other neck issues, shoulder pain (tendonitis), other shoulder issues, rotator cuff, tennis elbow, golfer’s elbow, wrist tendonitis, writs tenosynovitis, carpal tunnel syndrome (CTS), herniated disc, other lumbar disc issues, knee osteoarthritis, knee injury, hip arthritis, plantar fasciitis, other such as back pain or thumb pain

*Logistic regression; effect likelihood ratio test

**Fig. 4:**
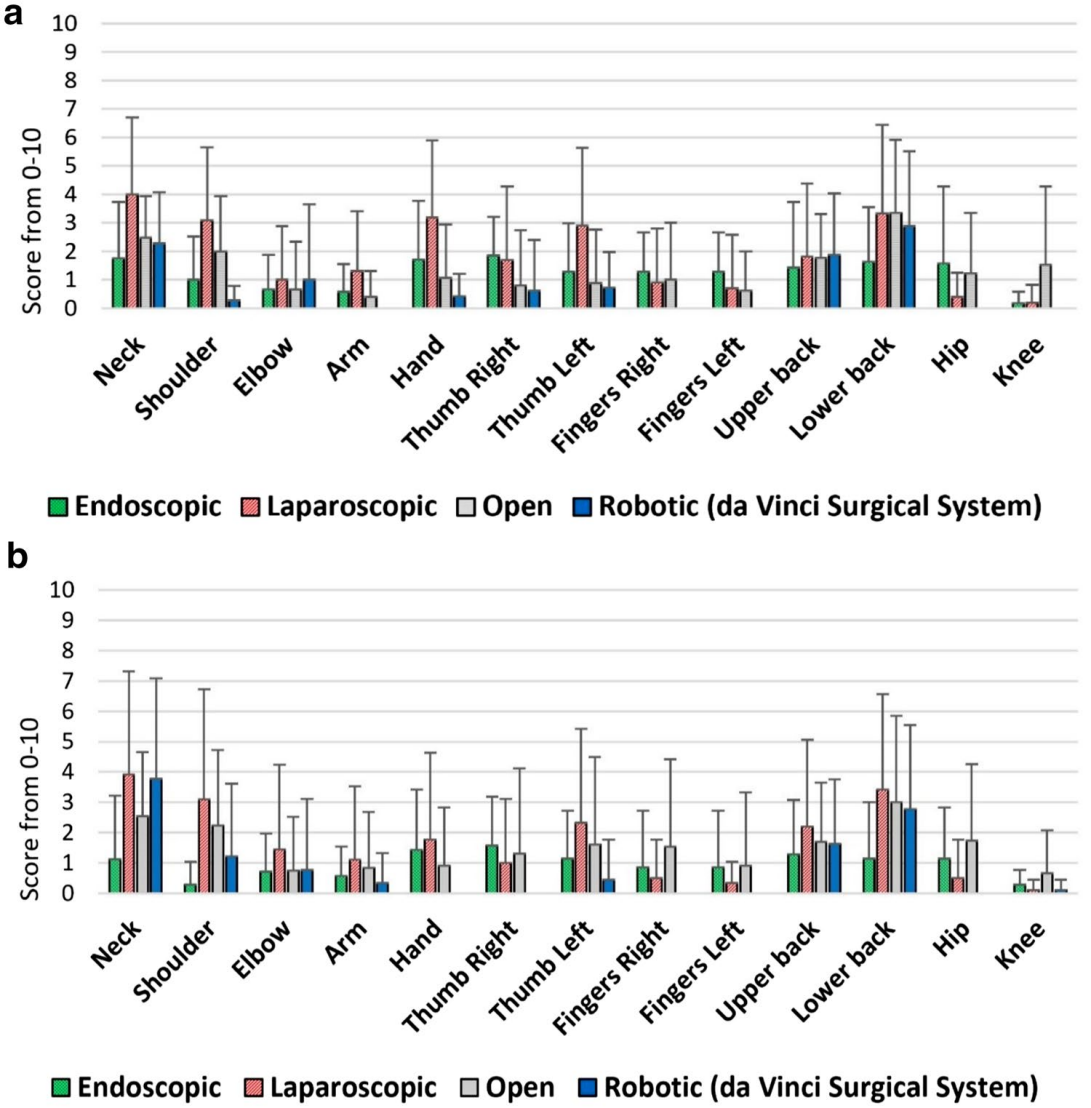
**a** Typical discomfort or pain immediately after surgery over the past 30 days rated from 0 = none to 10 = as bad as you can imagine. **b** Worst discomfort or pain in the past seven surgical days rated from 0 = none to 10 = as bad as you can imagine

The intraoperative body part discomfort or pain were recorded more granularly through two questions as “typical discomfort or pain immediately after surgery over the past 30 days” (Fig. 4a) and “worst discomfort or pain in the past 7 surgical days” (Fig. 4b) (all rated from 0 = none to 10 = as bad as you can imagine).

Shoulder discomfort or pain immediately after surgery over the past 30 days was significantly lower for robotic surgeons compared to laparoscopic (*p* = 0.0174) and open (*p* = 0.0377) surgeons. Additionally, left hand fingers discomfort or pain immediately after surgery over the past 30 days was lower for robotic surgeons compared to endoscopic surgeons (*p* = 0.0109). Surgical modality did not show a significant effect on the other body part scores and thus, pairwise comparisons were not performed (Fig. 4a and b).

Surgeons’ upper extremities are utilized to perform surgery at the sharp end; thus, a specific question was asked- “During the past week, were you limited in your work or other regular daily activities as a result of your arm, shoulder, or hand problem?” with a “yes/no” response to five options of “Arm, shoulder, or hand pain at rest”, “Arm, shoulder, or hand pain when performing any specific activity”, “Tingling (pins and needles) in your arm, shoulder, or hand”, “Weakness in your arm, shoulder, or hand”, “Stiffness in your arm, shoulder, or hand”. The “yes” response to at least one of the five options versus “no” to all five options were compared among the four surgical modalities (endoscopic = 20%, laparoscopic = 47%, open = 15%, and robotic = 7%; at least one yes response) and laparoscopic surgeons were associated with more upper extremity symptoms compared to open (*p* = 0.0363) and robotic (*p* = 0.0360) surgeons.

**Fig. 5:**
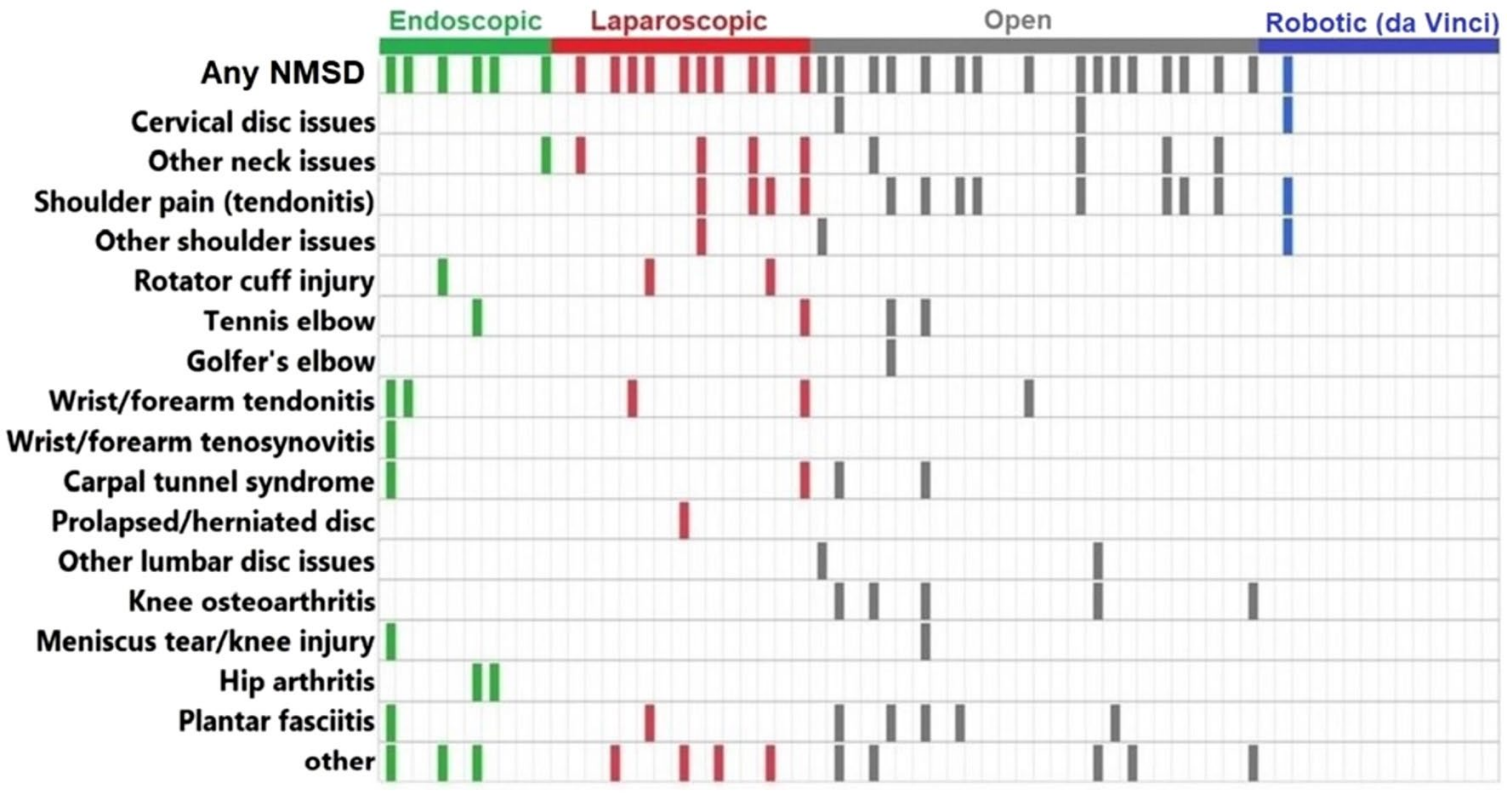
Neuromusculoskeletal disorders (NMSD) among 65 surgeons (10 endoscopic, 15 laparoscopic, 26 open, and 14 robotic surgeons). The “other” included “not specified”, “sacroiliac joint pain”, “foot pain”, “low back pain”, “sacroiliitis”, “back pain”, “thumb pain”, “ulnar nerve entrapment”, “hand arthritis”, “trigger finger”, “thumb joint arthritis”, and “back pain”, respectively according to the graph

Finally, NMSDs among the 65 surgeons allocated to the four surgical modalities have been presented in Fig. 5. It should be clarified that a new variable was defined as “Any NMSDs” and if the surgeon chose “Yes” for at least one symptom among all the NMSDs in the question, this variable (Any NMSDs) was equal to “Yes” for that participant (noted in Fig. 5). The prevalence of NMSDs was 60% among endoscopic, 67% among laparoscopic, 62% among open, and 7% among robotic surgeons (51% for the 65 surgeons with a dominant modality and 49% for the total 79 surgeons). The occurrence of at least one NMSD was significantly lower among robotic surgeons compared to the other modalities (all *p*-values < 0.05) (Table 2); however, the results of the logistic regression models did not show any significant differences in the specific NMSDs among the four modalities.

## Discussion

This study compared surgeons’ workload, physical discomfort, and NMSDs among four surgical modalities (endoscopic, laparoscopic, open, and robotic). Several surgical specialties (urologic, gynecologic, breast, thoracic, and general surgeons) were included, and a comprehensive survey was used. The aggregated findings suggest that robotic surgery is the most physically ergonomic surgical modality followed by endoscopic surgery, while either open or laparoscopic surgeries could be ranked as the least physically ergonomic surgical modality. Additionally, the results highlight the need for better ergonomics even in robotic and endoscopic surgeries, as they are also associated with pain, discomfort, and neuromusculoskeletal symptoms.

Physical demand was the highest for open surgery and the lowest for endoscopic and robotic surgeries, and consistently, physical discomfort and pain after a day of surgery was the highest for open surgery while not significantly different among the other three modalities (all *p* < 0.05) (Fig. 2 and Table 1). Furthermore, open and robotic surgeries required the highest levels of mental workload followed by laparoscopic (not significantly different from robotic) and endoscopic surgeries, respectively (all *p* < 0.05). A contributing factor in the lower levels of mental demand in endoscopic surgical procedures could be the shorter, less complex, and limited type of surgeries that can be performed endoscopically. Additionally, the high level of mental demand in robotic surgical procedures may be partially due to the novelty of this surgical modality (known as the learning curve), especially with the newer da Vinci SP surgical system [30]; however, it does not guarantee that the high level of mental demand will be completely resolved by gaining more experience [30].

There is not a universally endorsed threshold for workload regarding surgeon’s health and performance; however, the scores over 10–11 out of 20 (over 50–55%) for workload have been used in previous literature [31–33]. These thresholds highlight the high levels of physical workload for open and laparoscopic surgeries, high levels of mental workload for open, robotic, and laparoscopic surgeries, and high levels of physical pain and discomfort for all four surgical modalities. These findings are consistent with previous literature that have reported robotic surgery to be a more physically ergonomic surgical modality for the surgeon compared to open [21–23] and laparoscopic [21–24] surgeries, while it still needs to be enhanced ergonomically to minimize the risk of developing NMSDs [21–23, 27].

Baseline pain before surgery was reported by 29% of the surgeons while past or current pain was reported by about 62% of the surgeons. These results are within the range compared to two recent systematic reviews and meta-analyses studies which reported surgeons’ pain prevalence of about 35–70% [1, 2]. While no significant difference was found among the four surgical modalities for the baseline pain before surgery, pain (past or current) was lower for robotic surgeons compared to other surgical modalities (all *p* < 0.01), which provided additional evidence that robotic surgery could be considered a more physically beneficial surgical modality for surgeons compared to open, laparoscopic, and endoscopic surgeries.

Body part discomfort or pain (immediately after surgery over the past 30 days) was lower in the shoulder for robotic surgeons compared to laparoscopic and open surgeons and in left hand fingers for robotic surgeons compared to endoscopic surgeons. However, the results show that robotic surgery could lead to relatively high pain and discomfort scores especially in neck, upper back, and lower back (Fig. 4a and b). This is consistent with previous literature that underlined the neck and trunk as body parts potentially at risk during robotic surgery [26, 27, 34] while reported finger symptoms associated with robotic surgery [23, 27] was not confirmed through our study. Despite the fact that the da Vinci surgeon console provides the ability to adjust the ergonomic positions of the stereo viewer, arm rest, and foot controls, improper setup of these settings may contribute to neck and trunk symptoms reported. This highlights the importance of taking the time to properly set up the console for optimal ergonomic experience. Hokenstad et al. suggested a protocol for the da Vinci surgeon console platform to make ergonomic adjustments and reported improved postures at surgeons’ neck and right upper arm [35]. More investigation is required to quantify the ergonomic impact of these adjustments on mitigating surgeons’ intraoperative body part discomfort, fatigue and in the long run, work-related NMSDs.

The symptoms related to upper extremities (in the past week) were significantly higher in laparoscopic surgeons compared to open and robotic surgeons. This agrees with a pilot study that reported higher shoulder discomfort and worse ergonomic postures in upper arm, lower arm, and wrist during laparoscopic gastric bypass surgery compared to the robotic approach [26]. However, these findings do not confirm the results of a different pilot study that showed lower muscle activations in upper body muscles during the laparoscopic portion of sigmoid colectomy compared to open section of that procedure [19]. It is noteworthy that our study compared the surgical modalities generally while these previous studies have compared surgical modalities during a specific surgical procedure or different sections of the same procedure which limits the generalizability of their findings.

Self-reported NMSDs was as high as ~ 50% in our study population, strongly emphasizing the need for ergonomic interventions in the OR. NMSDs have been found to be the most common health problem reported by surgeons (general, colorectal, vascular, and cardiothoracic) that led to retirement [6]. Experts in this research area (e.g., health care ergonomists) should dedicate more effort to the development and evaluation of potential interventions such as ergonomic education [10, 36, 37], intraoperative stretching microbreaks [11–14], and exercises before and after the surgical procedures [10] to mitigate surgeons’ body part discomfort/pain and the risk of developing NMSDs. Furthermore, exploring the NMSDs among four surgical modalities showed that its prevalence was significantly lower in robotic surgeons (7%) compared to the other surgical modalities (between 60 and 67%). These findings provide more evidence in support of robotic surgery as a physically beneficial approach for surgeons compared to the other surgical modalities. Additionally, illustrating the specific NMSDs by particular surgeon by surgical modality (Fig. 5) suggested that NMSDs among the 65 surgeons of this study (1) usually affected surgeons in more than one body part, (2) affected spine (either cervical or lumbar) over all surgical modalities, (3) affected different body parts for endoscopic, laparoscopic (relatively less NMSDs in lower extremities), and open surgeons, and (4) had a more focused distribution among the robotic surgeons (neck and shoulders).

This study has several limitations. While the response rate of 32% provided us with 79 surgeons overall and 65 surgeons with a dominant modality, this relatively low-rate response may have led to a skewed data set toward the surgeons who were facing high workload, body part pain/discomfort, or NMSDs. Additionally, a subjective evaluation through a survey is always prone to inaccuracies such as recall bias. The dominant surgical modality and neuromusculoskeletal symptoms were all based on the surgeons’ responses. Despite these limitations, it is noteworthy that the results of this study include surgeons from three hospitals in three states and a distributed health system with both complex and routine cases. The next phase of this study will aim to use surgeons’ electronic medical records to extract their NMSDs while their workload per surgical modality will be obtained through review of surgical practice records retrospectively for a period of 5–10 years. Additionally, despite our diligent work, the sample size of the data was small or imbalanced especially when the data were stratified for some statistical analyses which could be the underlying reason for the few statistically significant differences between the surgical modalities. However, it is expected that the next phase of the study will lead to a greater sample size as the surgeons are not required to complete a survey. Finally, there are other confounders that may affect surgeons’ physical condition such as non-surgical daily activities (e.g., sports, playing music, etc.). They were assumed to be randomly distributed among the four surgical modalities, not affecting the results of comparing the four surgical modalities, which was the focus of this study.

## Conclusions

It could be concluded that laparoscopic and open surgeries were associated with higher physical demand, and end of operative day physical pain/discomfort (only open) compared to endoscopic and robotic surgeries. Mental demand was the lowest for endoscopic surgery while relatively high for the other three surgical modalities. Having neuromusculoskeletal pain (past or present) and the occurrence of NMSDs was lower in robotic surgeons compared to the other surgical modalities. Overall, robotic surgery was found to be physically beneficial to the surgeon; however, it still warrants ergonomists’ attention to enhance the physical aspects of this surgical modality and reduce its required mental workload. It is essential to dedicate more studies to the development and evaluation of potential ergonomic interventions to mitigate surgeons’ body part discomfort/pain and the risk of developing NMSDs.
